# Machine learning analysis of population-wide plasma proteins identifies hormonal biomarkers of Parkinson’s disease

**DOI:** 10.3389/fnagi.2026.1730550

**Published:** 2026-03-10

**Authors:** Fayzan Chaudhry, Tae Wan Kim, Olivier Elemento, Doron Betel

**Affiliations:** 1 Tri-Institutional PhD Program in Computational Biology, New York, NY, United States; 2 Institute for Computational Biomedicine, Weill Cornell Medicine, New York, NY, United States; 3 Division of Hematology, Department of Interdisciplinary Engineering, Daegu Gyeongbuk Institute of Science and Technology, Daegu, Republic of Korea; 4 Caryl and Israel Englander Institute for Precision Medicine, Weill Cornell Medicine, New York, NY, United States; 5 Division of Hematology and Medical Oncology, Department of Medicine, Weill Cornell Medicine, New York, NY, United States

**Keywords:** biomarkers, deep learning, neurodegenerative disease, Parkinson’s disease, proteomics

## Abstract

With the number of Parkinson’s patients expected to rise due to an aging population, there is an increasing need to identify new diagnostic markers. These markers should be affordable and suitable for routine use to monitor the population, help stratify patients for treatment pathways, and provide new avenues for therapy. Genetic predisposition and familial forms account for approximately 10% of Parkinson’s disease (PD) cases, leaving a large fraction of the population with minimal effective markers for identifying high-risk individuals. The establishment of population-wide omics and longitudinal health monitoring studies provides an opportunity to apply machine learning approaches to these unbiased cohorts to identify novel PD markers. In this study, we present the application of three machine learning models to identify protein plasma biomarkers of PD using plasma proteomic measurements from 43,408 UK Biobank subjects as the training and test set and an additional 103 samples from the Parkinson’s Progression Markers Initiative (PPMI) as external validation. We identified a group of highly predictive protein plasma markers, including known markers Dopa decarboxylase (DDC) and Calbindin 2 (CALB2) as well as new markers involved in the JAK–STAT and PI3K-AKT pathways and hormonal signaling. We further demonstrated that these features are well correlated with UPDRS severity scores and stratified these into protective and risk-associated features that potentially contribute to the pathogenesis of PD.

## 1 Introduction

Parkinson’s disease (PD) is the second most common neurodegenerative disease, with an estimated prevalence of 10 million people worldwide (Ball et al., 2019). There is a massive economic burden on individuals, families, and the U. S. government, totaling over 50 billion dollars per year (Yang et al., 2020), and the number of PD patients is expected to grow with increased longevity of the general population (Bach et al., 2011). Parkinson’s disease not only diminishes the quality of life but also imposes a substantial societal burden through caregiving needs, lost productivity, and high healthcare costs. PD symptoms are marked by tremors, bradykinesia, and other movement-centered symptoms. This condition can eventually lead to severe loss of basic movements paired with swallowing difficulties. Pathologically, the disease is marked by the death of midbrain dopamine (mDA) neurons in the substantia nigra. This extensive death of mDA neurons in the substantia nigra creates Lewy bodies that impair cell function until death (Roeters et al., 2017) and results in the release of protein products into the serum and cerebrospinal fluid (CSF) (Santacruz et al., 2021). Symptoms often lag disease pathology, and the presymptomatic (prodromal) phase can last approximately 20 years (Roos et al., 2022). The onset of symptoms occurs after the majority of dopamine neurons are lost, presenting a significant challenge for early intervention and treatment options (Mandybur, 2018). A9 neurons are the mDA subtype with the highest loss in PD. Therefore, an increase in blood plasma levels of specific A9 proteins could be strong candidate biomarkers for early detection (Kamath et al., 2022). Single-cell analyses of A9 neurons lay a foundation that could provide deep insights into PD (Ma and Lim, 2021), but they are costly and are not currently used for diagnostics. Due to the long prodromal phase of PD, early detection is essential for intervention therapy (Mahlknecht et al., 2022; Hawkes, 2008). High-throughput proteomic screens allow for fast and cheap identification of impactful proteins. These new minimally invasive blood tests can be widely used in the aging population to diagnose conditions and provide early, targeted treatment, unlike CSF testing or imaging, which are often costly or too invasive.

Previous studies of PD biomarkers have highlighted a strong inflammatory component in PD, including a heavy effect size of dipeptidyl peptidase 7 and granzyme B (Rutledge et al., 2024). Recent evidence has shown that inflammation may contribute to the worsening of PD condition and enhance disease progression through reactive oxygen species that mDA neurons are particularly susceptible to Stojkovska et al. (2015). In addition to inflammatory markers, hormonal markers such as estradiol and testosterone have been shown to have significant correlations with PD progression metrics (Bovenzi et al., 2023), and lower incidence of PD in women may be partially attributed to the protective effect of estrogen (Shulman, 2002).

Previous studies to understand PD risk centered on genetic information using variations of linear models (Escott-Price et al., 2015; Han et al., 2020). Genome-wide association studies (GWAS) are based on the idea that many single-nucleotide polymorphisms, which individually have minor associations with PD, would compound and lead to stronger predictive models for developing PD (Euesden et al., 2015). Nalls et al. have obtained 0.69 area under the receiver operating curve (AUROC) PD classification performance from variant-only models trained on the UK Biobank (Nalls et al., 2019). Previous research using risk models estimates the probability of developing PD over time, helping to identify individuals at high risk for preventive care. Our current study focuses on developing early diagnostic models that diagnose or categorize individuals as having PD or not, aiding in therapeutic decision-making. More recent approaches such as whole genome sequencing, proteomics, or phenotypic evaluation can add significant predictive values. CSF proteomic models have shown strong classification of PD but are invasive and carry some risk of complications, which would exclude their use for large-scale screens (Kwon et al., 2022; Williams and Fost, 1992). More recent methods incorporate machine learning approaches for classification of PD featuring multiomic models spanning transcriptomic (Makarious et al., 2022) to in-depth invasive CSF (Tunbridge et al., 2019), sensor (Um et al., 2017), and imaging data (Tsukita et al., 2022).

The multiomic models presented here use high-throughput proteomic data generated by Olink assays, demographic information, and genetic data. The proteomic data are obtained using minimally invasive and low-cost information using only 1 μL of plasma (Zhang et al., 2024). This information will become more widespread in the future and is ideal for non-invasive combinations for screening and understanding the mechanism of disease pathology.

Using proteomic data across large populations can aid the discovery of more disease insights. Neuronal markers that may leak into CSF and then blood have been shown in studies focused on CSF in limited sample sizes (Phani et al., 2012; Winchester et al., 2022). Proteomic data have been collected from the substantia nigra to identify differences in pathways between PD cases and controls (Jang et al., 2023). Previous state-of-the-art CSF proteomic models for PD prediction yielded an AUROC of 0.80 for held-out sub-cohorts, and combining CSF and plasma proteomics yielded an AUROC of 0.89 (Rutledge et al., 2024; Phani et al., 2012). However, CSF and imaging measurements are not routine and are typically collected from high-risk or diagnosed PD patients, which are less applicable for population screens (Um et al., 2017). Furthermore, it is not clear whether these biomarkers are correlated with the severity of PD, as defined by the Unified Parkinson’s Disease Rating Scale (UPDRS), such that they can be used to track disease progression.

We used a deep learning model, a regression model, and a support vector machine (SVM) model to identify potential novel biomarkers for PD. Our results identified that 9 of our top 20 features from the neural network are hormonally related. We characterized these proteins, such as prolactin (PRL), through protein–protein interaction queries as well as through extensive computational analysis and literature reviews. Additionally, we found enrichment for the JAK–STAT pathway, which plays a strong role in immunity as well as other functions. We further validated some of our top features identified in the UK Biobank on an independent external validation set (PPMI). Following this, we used top features to determine PD severity through the UPDRS score, canonically accepted as a severity metric for PD. To the best of our knowledge, this is one of the largest population-based proteomic studies for Parkinson’s disease, leveraging machine learning approaches. Furthermore, we validated the identified biomarkers by correlating them with Parkinson’s disease severity scores, demonstrating their potential utility for tracking disease progression.

## 2 Results

### 2.1 Study design overview

**Figure 1:**
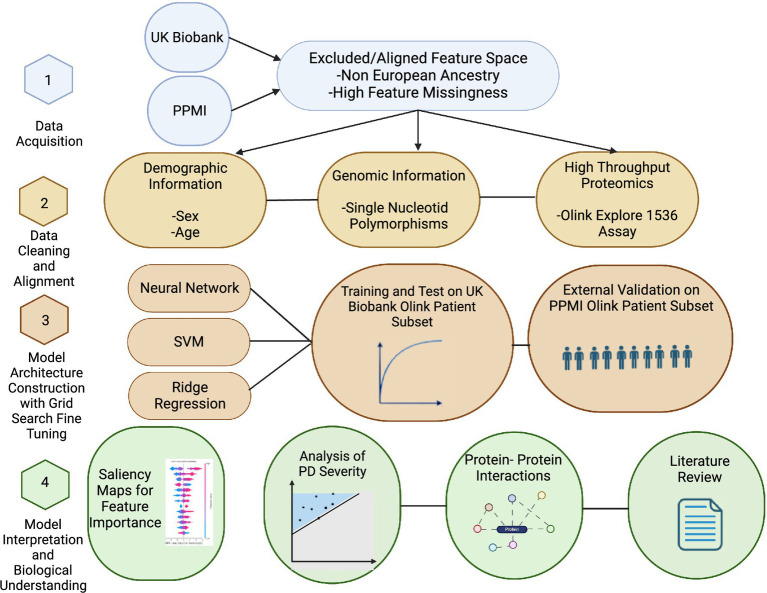
Outline of the study. The study is composed of four steps. In the first step, data QC and filtering were performed. In the second step, serum proteomic Olink data and SNP polymorphism array features from the UK Biobank and PPMI were matched to a common subset. These features were used in ridge regression, support vector machine, and neural network models to predict PD cases in the third step. The last step included biological interpretation of the features and correlation with PD severity scores.

This study was performed in four stages: data acquisition, data cleaning plus alignment, model architecture tuning, and model interpretation (Figure 1). Data were acquired with permission from the UK Biobank and PPMI resources (Biobank, U, 2014; Aleksovski et al., 2018). The two studies used different approaches and technologies for variant detection. The second step consisted of extensive alignment of genomic array results, followed by filtering to match the PPMI genomic feature set to that of the UK Biobank. We developed three models—a neural network, support vector machine (SVM), and ridge regression—to classify PD vs. control using the proteomic data, genetic variants, or a combination of both. Training and testing of the model were performed on the UK Biobank datasets, and external validation was performed on the PPMI data. Model interpretation was performed by extensive literature review of the top features and KEGG pathways to ascertain biological interpretation. To identify the most predictive features, Shapley Additive Explanations (SHAP) (Lundberg and Lee, 2017) were calculated for the top 85 (12.5%, or approximately 250 top features from the top two models) common features between the two top-performing models. These features were used in a linear model to predict the PD severity scores defined by the Unified Parkinson’s Disease Rating Scale (UPDRS) metric. The Unified Parkinson’s Disease Rating Scale (UPDRS) comprises multiple subscales that capture distinct clinical domains of Parkinson’s disease, including non-motor experiences of daily living (Part I), motor experiences of daily living (Part II), motor examination (Part III), and motor complications (Part IV). Among these, the UPDRS III Motor Examination is widely regarded as the most sensitive to disease progression and typically demonstrates the fastest longitudinal change, reflecting worsening motor impairment.

### 2.2 Dataset populations

In this study, we took advantage of two large population cohorts. The first is the UK Biobank, which is one of the largest biomedical databases that contains genomics and health-related data from half a million UK residents (Bach et al., 2011; Sudlow et al., 2015). The second cohort is patient samples from the Parkinson’s Progression Markers Initiative, an observational study sponsored by the Michael J. Fox Foundation aimed at identifying biomarkers to predict, diagnose, and track Parkinson’s disease progression (Marek et al., 2011). Both study populations are a majority of European ancestry and are skewed toward older patients with a range of 31–83 years and median ages of 59 years and 60 years for the UK Biobank and PPMI, respectively (Table 1). Selecting UK Biobank participants who were profiled by the Olink Explore 1,536 protein panel reduced the number of patients to 43,408. Among those with Olink data, 778 patients were diagnosed with Parkinson’s disease, resulting in a case-to-control ratio of 2%. The UK Biobank was used predominantly to train and benchmark the predictor algorithms, whereas the 103 PPMI samples were used as an independent external validation dataset. The filtered PPMI dataset had a case-to-control ratio higher than the UK Biobank of approximately 24%. This class imbalance is common in a disease setting. Thus, distributions of cases were different, which impacted performance on the PPMI validation. This is primarily due to the PPMI data selecting for PD patients, while the UK Biobank is a population-level study. Deep learning models can be ill-suited for modeling rare case events due to overfitting (Bejani and Ghatee, 2021). Thus, methods were used to overcome this, including dropout, mini-batches, class weighting, and optimized learning rates.

**Table 1:** Demographics and feature space of UK Biobank and PPMI cohorts profiled with the Olink Explore protein panel.

Characteristic	UK Biobank	PPMI
Unique patients	43,408	103
Male	20,079	74
Female	23,329	29
Age (minimum, medium, and maximum)	40, 59, 70	31, 60, 83
Parkinson’s disease	778	26
Controls	42,630	77
Proteomic serum	1,463 proteins	1,463 proteins
SNPs	Axiom Array	NeuroX Array and Immunochip Array

In terms of gender split, the UK Biobank has 54% female, and the PPMI has 28% female. Sex bias may influence the biomarkers studied, such as PRL and growth hormone (GH1), which are generally more abundant in women (Thapa and Bhusal, 2023; Span et al., 2000).

Another important difference between the two cohorts is their treatment status. The PPMI is selected for levodopa-naïve patients, indicating that the majority of the PD patients have not received dopaminergic therapy yet. This gives a proteomic profile of the disease state without the confounding effect of treatment. In contrast with the UK Biobank, roughly 4%, or 30 of 778 PD patients, of the PD patients in the UK Biobank are on levodopa or similar medications (levodopa, co-careldopa, Sinemet-LS tablets, Half-Sinemet CR M/R tablets, and Sinemet-62.5 tablets). This treatment, although small, is a limitation that can alter proteomic profiles and confound biomarker identification and model performance. Currently, levodopa is known to impact DDC levels, which was one of the features identified in our study (Paslawski et al., 2023).

### 2.3 Multimodal proteomic and genetic models

**Figure 2:**
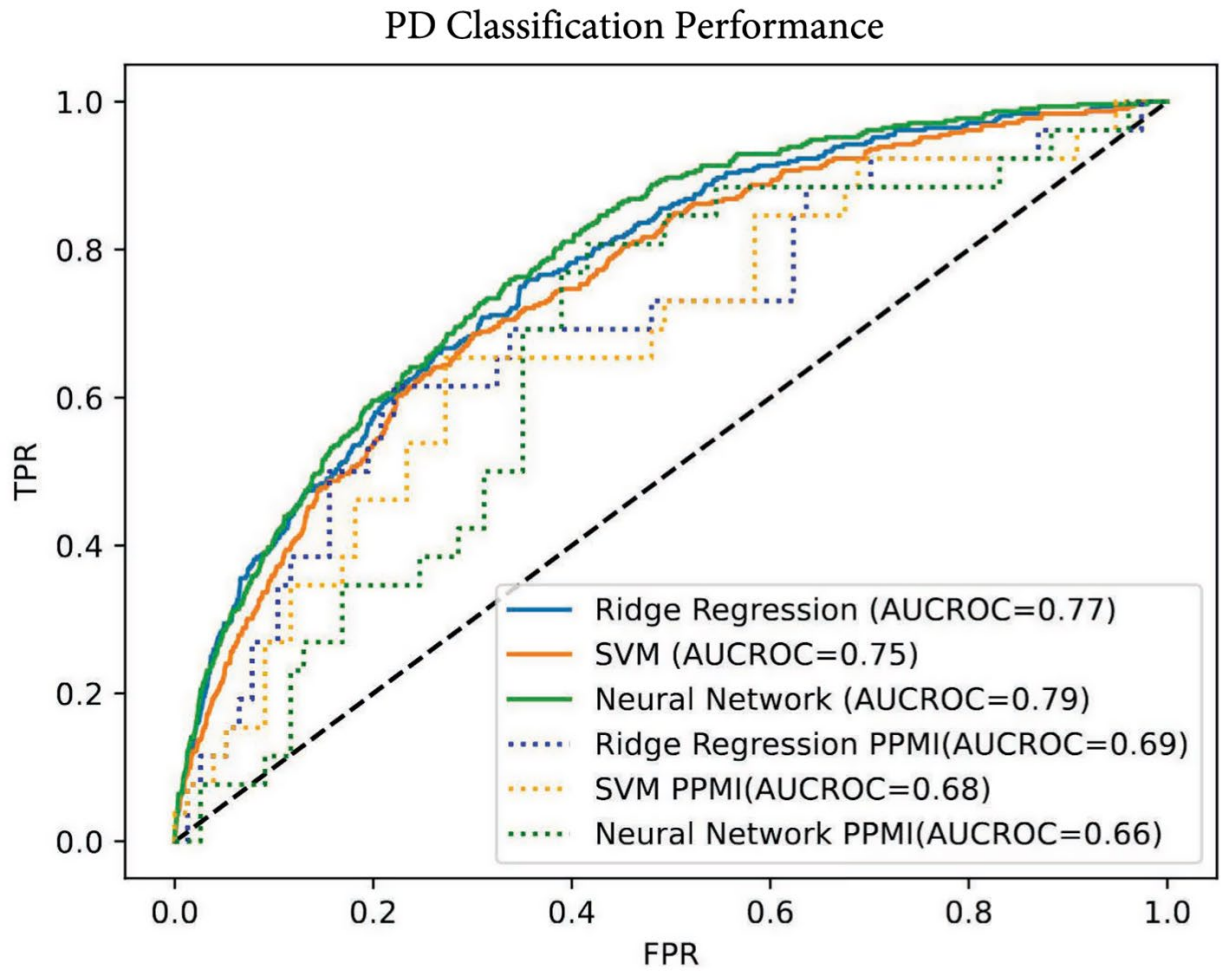
AUC-ROC classification performance. AUC of neural network, baseline ridge regression, and support vector machine models on the UK Biobank held-out test set and PPMI validation set. The solid lines represent the test set, and the dotted lines represent the external validation on the PPMI dataset.

Using the subset of harmonized genomic variants and proteomic data, we developed three computational models to classify PD status for individual subjects. We used neural network, SVM, and ridge regression models that input proteomic and variant profiles and produce a probability value for PD diagnosis that is evaluated by the ROC analysis of classifier performance (Figure 1). Neural networks (NN) are a general framework for estimating complex, non-linear functions, making them ideal for capturing intricate relationships in high-dimensional data. Support vector machines (SVM) identify a dividing hyperplane to maximize class separability, while ridge regression is a linear model that uses a penalized linear combination of features to mitigate overfitting. The area under the receiver operating curve (AUC-ROC) measures were comparable across all three models on both the training UK Biobank and the validation PPMI datasets (Table 2; Figure 2). All three methods had comparable performance of ~0.77 AUC-ROC on the held-out test data from the UK Biobank, with the NN having the highest performance of 0.79 (Figure 2). The three models performed similarly on the PPMI external validation with an AUC-ROC of ~0.67. The performance drop on the external validation set is potentially due to differences in serum data capture and differences in case-to-control ratios of datasets. Additional factors impacting validation performance could be related to the PPMI being drug naïve vs. the UK Biobank having a larger number of patients under treatment. When considering sensitivity and specificity, the ridge regression model had the highest sensitivity at 0.76, while the SVM model had the highest specificity at 0.69. The neural network achieved the best overall balance, with sensitivity and specificity of 0.73 and 0.69, respectively. These metrics were both within 3% of the other models, despite a slight reduction in performance on these metrics.

**Table 2:** Performances of the multiomic prediction models.

Performance metric	Ridge regression	SVM	Neural network
AUC-ROC test set (UK Biobank)	0.77	0.75	0.79
Sensitivity	0.76	0.67	0.73
Specificity	0.64	0.70	0.69
AUC-ROC validation (PPMI)	0.69	0.68	0.66

The three models’ performance was evaluated by the AUC, sensitivity, and specificity measures for PD classification performance on the UK Biobank held-out data as well as AUC performance on the PPMI external validation set.

Overall, model performances are comparable on both the held-out test set in the UK Biobank and the validation PPMI test set. While the neural network had slightly better performance on the training data, it was lower than the other models in the validation set, suggesting potential overfitting. In contrast, the ridge regression model had better performance on the validation set (Table 2), which, through regularization, often trades off some amount of training accuracy for improved generalization.

A possible confounding factor is the prior Parkinson’s disease (PD) and Levodopa (L-DOPA) treatment history that the majority of PD patients receive. The PPMI cohort did not receive prior treatment, and of the 778 UK Biobank patients, 30 had records of L-DOPA treatment (4%). To investigate the impact of these cases on model performance, we retrained the ridge regression models, excluding the levodopa-treated patients from the UK Biobank training set, and found that AUC-ROC values remained similar at 0.76 in the test for the ridge regression model compared to 0.77 and 0.69 in external validation for the PPMI in both L-dopa naïve and original (Supplementary Figure 1). This suggests that the presence of levodopa treatment did not significantly impact the overall model performance.

### 2.4 Genetic-only model

**Figure 3:**
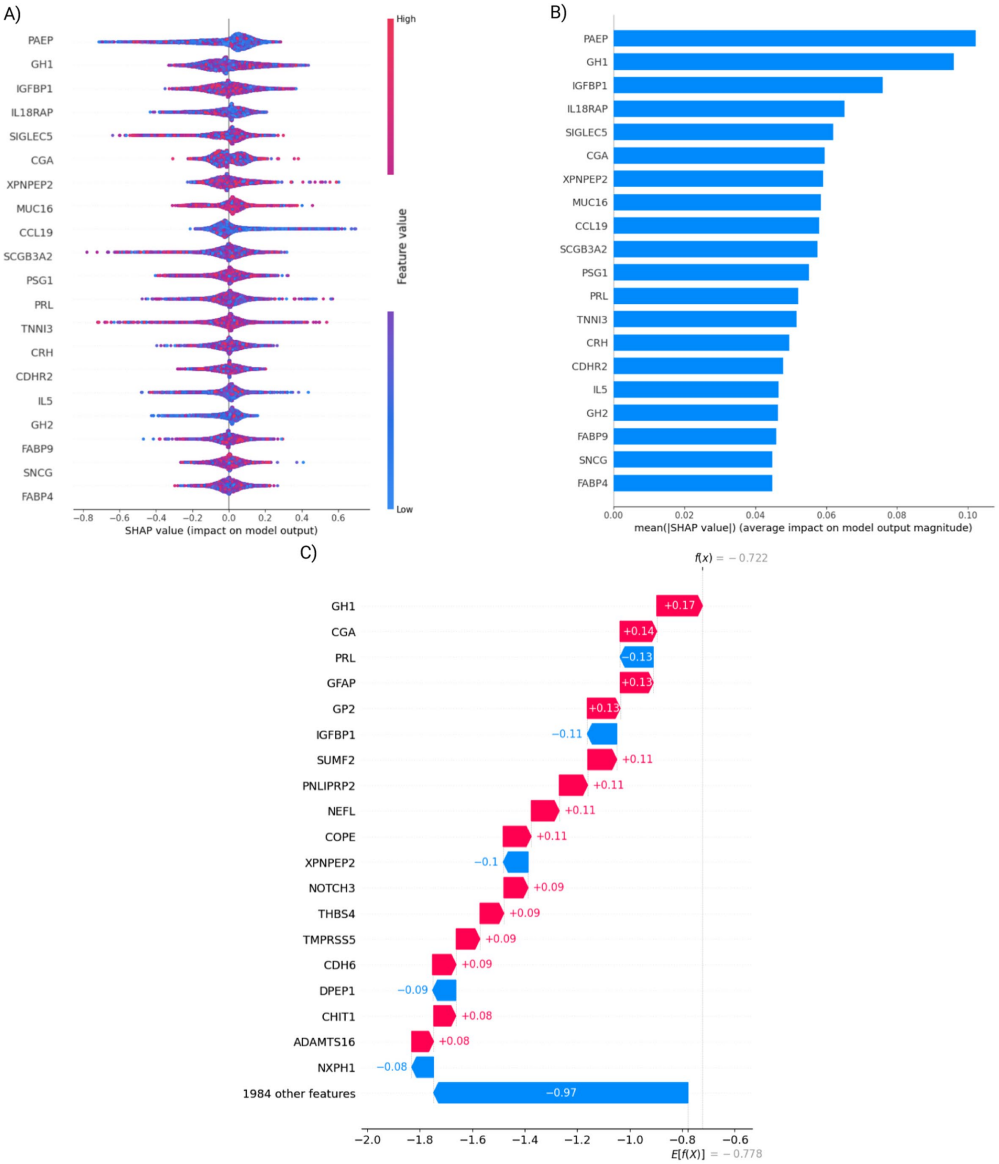
Evaluation of the top 20 neural network features. **(A)** SHAP value distributions of the top 20 neural network features. Each feature is represented by the distribution of SHAP values (i.e., impact on model output) over all test cases. Negative values represent negative weights for PD prediction, whereas positive values represent positive contributions to predicting PD. **(B)** The mean absolute SHAP values showing feature importance of the same 20 features. **(C)** A waterfall plot showing SHAP values, contribution to classification result for a single UK Biobank participant.

We created a variant-only model to analyze baseline performance without proteomic data that is akin to a polygenic risk score model. While all three models had reduced performance the neural network AUROC of 0.62 on the training set is significant reduction by over 25% relative to the multimodal model (Supplementary Table 1). This was expected, as no genetic variants appear in the top 20 feature set in the combined proteomic and single-nucleotide polymorphisms (SNP) neural network (Figure 3A). Although several genetic variants appeared in the top 85 of features, external validation performance on the PPMI cases of the variant-only model declined further to random performance of 0.50.

Overall, the use of only genotypic and demographic information, primarily sex and age, led to substantially reduced prediction performance relative to the multimodal modeling that includes the serum proteomic measurements. This is expected, as these features are typically used for polygenic risk scores to identify individuals with high risk, but their genetic penetrance is low. In contrast, proteomic data is a more immediate marker for PD state diagnostics.

### 2.5 Feature evaluation

We used saliency techniques to identify and better understand the most impactful features of both the Olink and SNP for the neural network. We investigated the magnitude of feature impact using the Shapley additive explanation (SHAP) score that quantifies feature impact on the model performance (Figures 3A,B). After investigating the top 20 features, we found neuronal-specific markers such as DDC and CALB2 that were expected outcomes, given that they are related to dopamine function (Devos et al., 2014; Mouatt-Prigent et al., 1994). Unexpectedly, we also identified hormonal markers such as prolactin and GH1, which are some of the most influential features. SHAP scores allow us to see individual scores from specific patients as well as groups of individuals, such as all women. For example, in one subject from the UK Biobank study, PRL’s negative value indicates a protective contribution, whereas GH1’s positive value indicates a harmful effect (Figure 3C).

**Figure 4:**
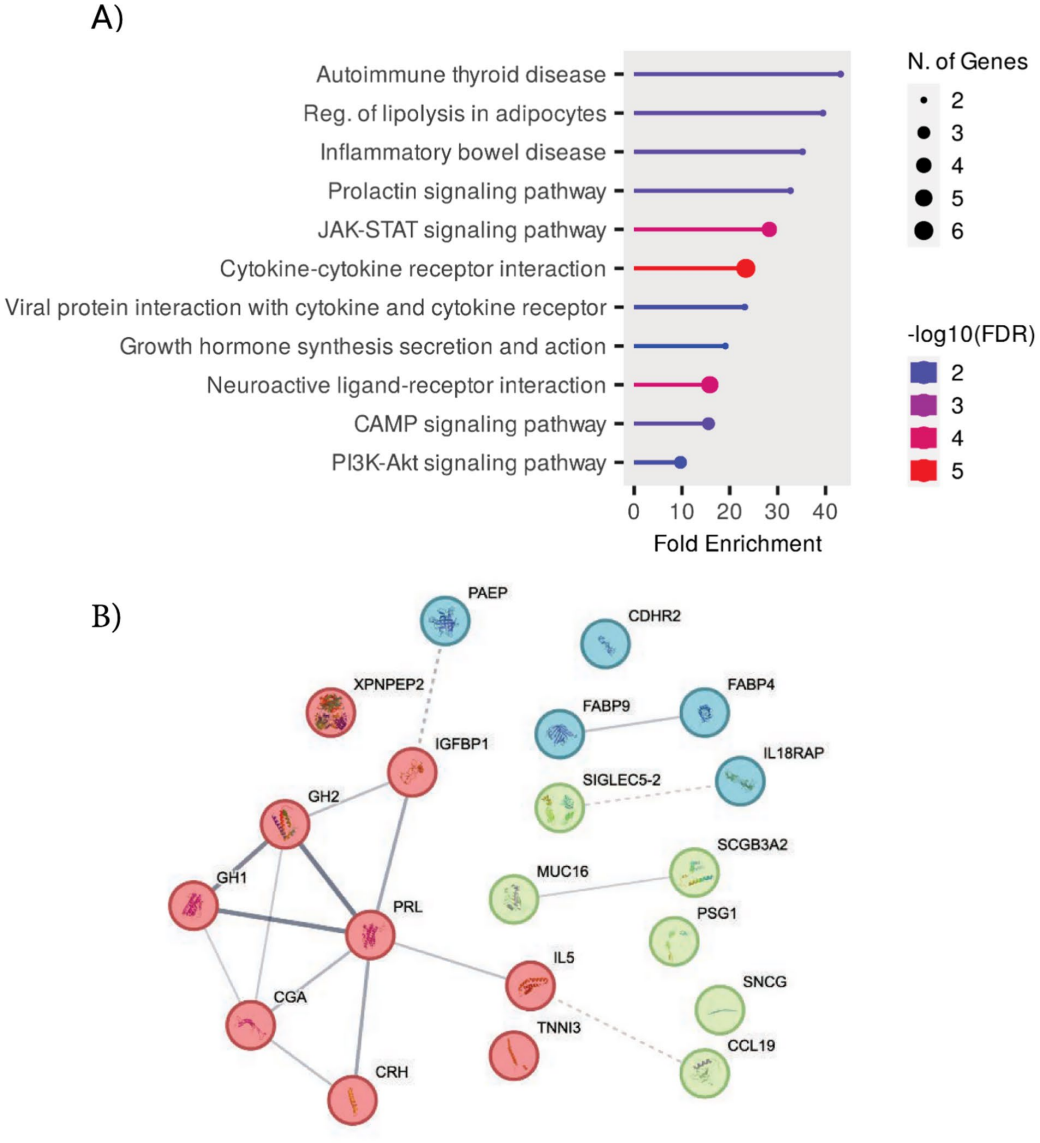
Top features functional analysis. **(A)** KEGG pathway analysis of the top features, based on the genes corresponding to the identified proteins, highlighting key biological pathways involved. **(B)** Protein–protein interaction (PPI) networks displaying the number of expected interactions of random proteins versus observed interactions. The PPI network exhibits significant enrichment with a *p* ≤ 6.15e-11. Three distinct clusters are shown in different colors, with the thickness of connections denoting the strength of connectivity across experimental data and literature data.

Further biological investigation through KEGG analysis of these top markers yielded strong enrichment for the prolactin signaling pathway and the JAK–STAT and PI3K-Akt pathways (Figure 4A). JAK–STAT and PI3k-Akt pathways were validated in other studies in which it was found that the PI3k-Akt pathway could be one of the most influential in a rapidly progressing PD subtype (Su et al., 2024). The hormonal signaling protein PRL, one of the most widely studied hormones, prolactin, also emerged as a top feature, suggesting involvement of a hormonal component to PD pathogenesis through the activation of JAK–STAT pathways and prolactin signaling pathways. Additionally, the protein–protein interaction enrichment test showed that six of the top features were interrelated hormones that are highly interconnected (*p* < 6.15 e-11) (Figure 4B; Franceschini et al., 2012). Our findings suggest that the JAK–STAT and PI3K-AKT pathways may act as central hubs in PD pathology, potentially linking neuroinflammation, hormonal signaling, and neuronal survival.

### 2.6 Feature correlation with PD severity score

**Figure 5:**
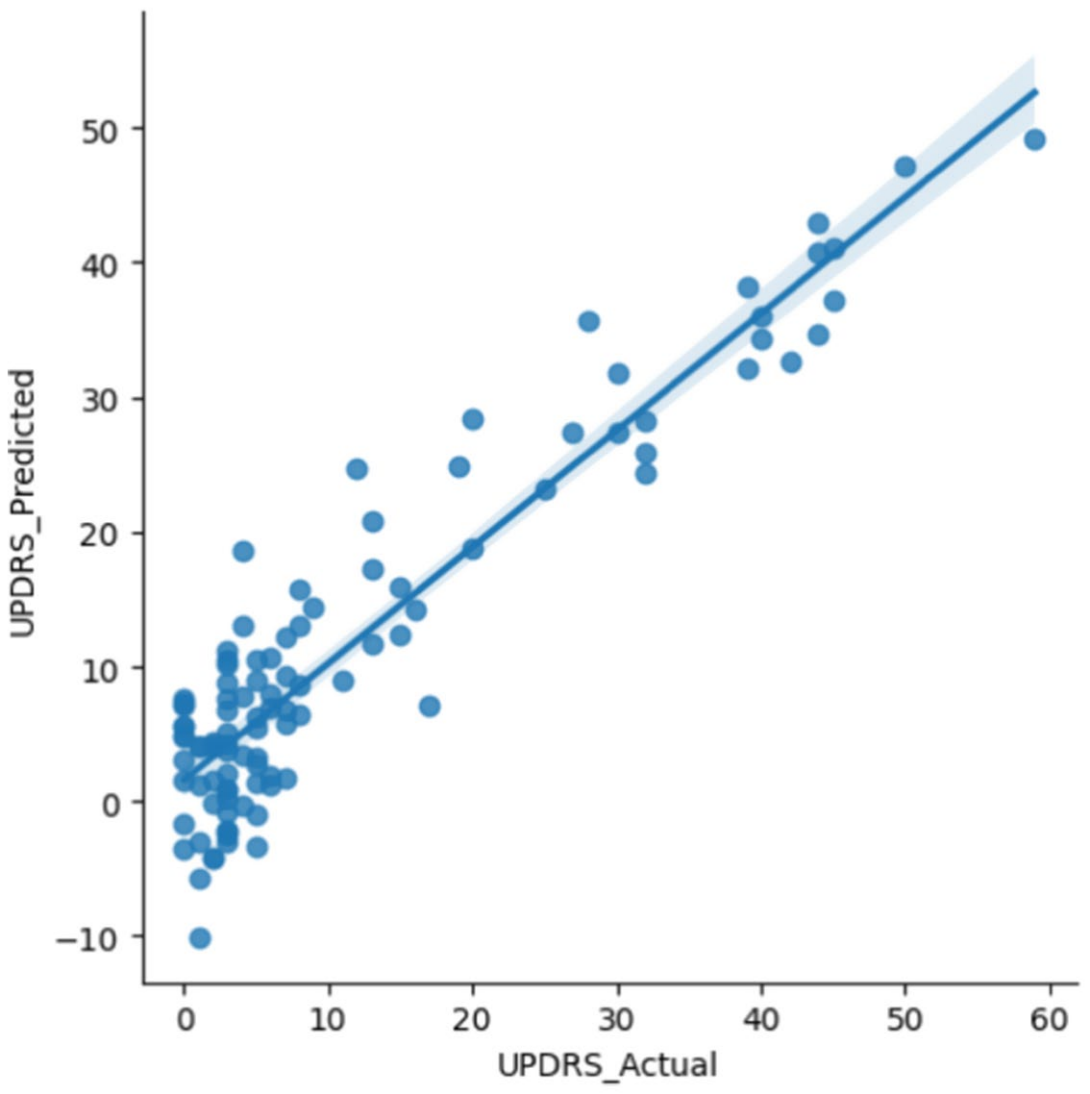
Linear model of UPDRS scores. The top 85 aligned features of the neural network and the ridge regression were used in the linear regression model to predict the UPDRS score on the PPMI validation set (*R*^2^ = 0.86).

We next investigated the shared features that the different models identified as significant to identify the core biological pathways that are predictive of PD. We subset the top 85 (12.5%) features that were common across the ridge regression and neural net models, excluding the SVM model due to slightly lower performance (see Methods section). These 85 common features were used to construct a linear regression model to predict the UPDRS score (Movement Disorder Society Task Force on Rating Scales for Parkinson's Disease, 2003), which is the accepted severity score for PD among physicians (Figure 5; Supplementary Table 2). We selected 85 features as the optimal number of descriptive features without overfitting based on empirical analysis of the coefficient of determination (R2) for various numbers of features across different adjusted R2 values (Supplementary Figure 2). We opted to use a straightforward linear regression model for interpretability and to investigate the relationship between model-derived PD classification scores and PD severity. While more complex predictive models may capture non-linear patterns with higher accuracy, the simplicity of the linear regression model facilitates clearer insights into how model outputs relate to clinical measures. This approach supports the broader aim of demonstrating that classification frameworks can produce outputs that meaningfully reflect disease severity in a clinically relevant and interpretable manner.

We found that these top features are well correlated with UPDRS values in the PPMI dataset (*R*^2^ = 0.86). Several SNPs were included in this top feature set as highly predictive of PD that were previously validated in the literature. These include rs12951632, rs11158026, and rs10748818, indicating their established association with Parkinson’s disease and further supporting their potential role in disease susceptibility and progression (Farrow et al., 2022; Fan et al., 2021). Two of the variants, rs12951632 and rs11158026, were identified in related genome-wide association studies as variants that are strongly associated with PD. The third variant, rs10748818, was previously identified as causing changes in the GBF1 gene, which is a critical factor in PD pathogenesis (Fan et al., 2021).

### 2.7 Feature signatures associated with increased PD

**Figure 6:**
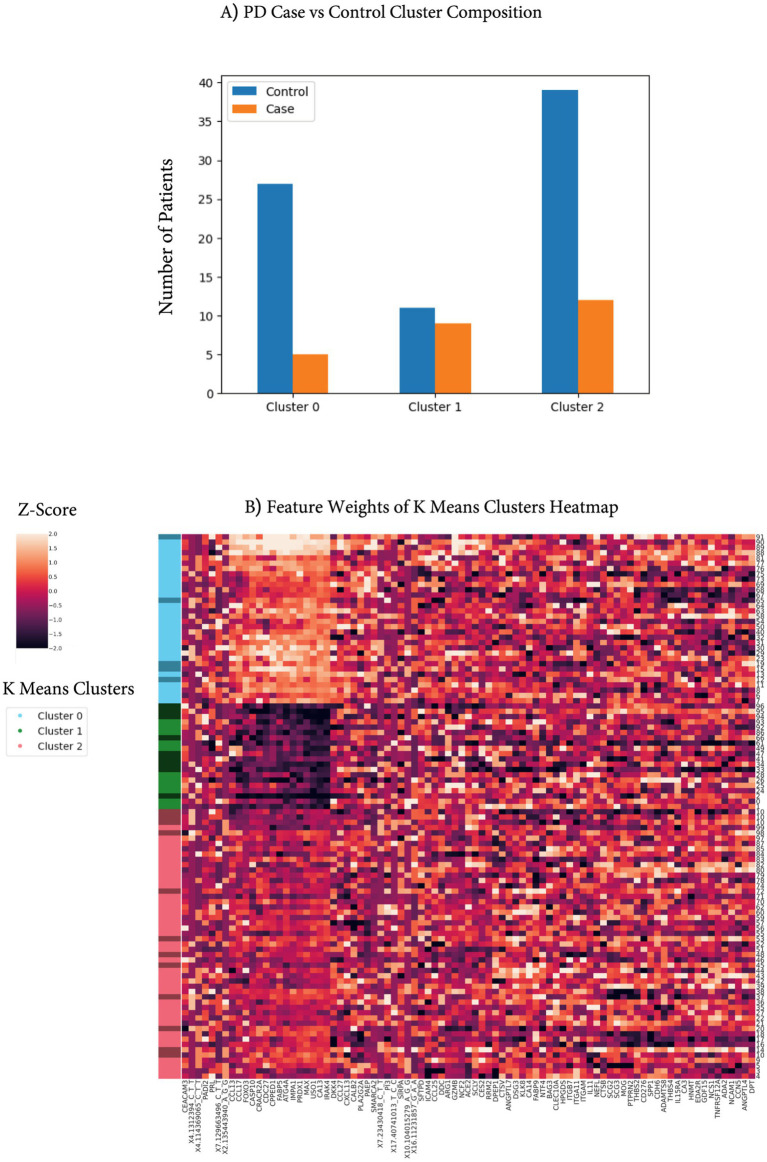
Feature clusters identify protective PD proteins. **(A)** KNN clustering of the top 85 features identified three predominant clusters. Bar plot highlighting the enrichment of PD relative to controls in Cluster 1. **(B)** Heatmap of protein level (Z-scores) expression in the PPMI datasets. The left-side color strip indicates cluster assignment (cluster 0 is blue, cluster 1 is green, and cluster 2 is red), where darker green, blue, or red indicates PD cases, while lighter colors indicate controls. Proteins are clustered to identify cluster signatures.

Next, we were interested in investigating the contributions of the features to the likelihood of PD. K-nearest neighbors clustering of the PPMI cohort using the shared 85 features identified three clusters of distinct signatures reflecting their underlying expression patterns. While 85 features were used to model UPDRS severity, patient clustering based on these 85 features was largely driven by 14 proteins that had varying degrees of expression. High abundance in cluster 0 was associated with low PD/control ratio, medium expression in cluster 2, and low expression in cluster 1 associated with high PD/control ratio (Figure 6A). Cluster 1 is strongly distinct from other clusters by a protein signature that is driven by 14 proteins that are negatively correlated with the PD (Figure 6B). One of these proteins is FABP5, a fatty-acid-binding protein, which was recently identified as a PD marker in an independent proteomic study of 99 PD patients (Hällqvist et al., 2024). In addition, a similar study by Hallqvist et al. used a mass spectrometry approach to identify protein markers from CSF and serum that predict PD in the prodromal phase among a small cohort (Hällqvist et al., 2024). While not all significant proteins from their study overlapped with the proteins measured in the Olink assay, four overlapping proteins (CST3, DKK3, PTGDS, and VCAM1) were found as significant PD markers in our study (adjusted *p*-value < 0.01, by *t*-test).

### 2.8 Feature correlations

To gain insights into the cross-feature correlation structure and specifically the 14 proteins predominant in the sample clusters, we performed a protein pairwise correlation analysis using UPDRS scores (Supplementary Figure 3). Two distinct, highly correlated protein groups were identified. The first group of proteins is characterized by cytokine–cytokine enrichment as well as involvement in the PI3K-AKT signaling pathway (Supplementary Figure 4A). This signature has a strong immune and inflammatory component, which has been a hallmark of PD (Ferrari and Tarelli, 2011; McGeer and McGeer, 2004), and every feature was positively correlated with UPDRS scores. The second group (marked in blue, Supplementary Figure 3) is associated with the MAPK signaling pathway as well as other apoptotic pathways (Supplementary Figure 4B). MAPK signaling has been strongly implicated in PD with connections to high-penetrance variants such as LRRK2 (Obergasteiger et al., 2018). This signature is similar to the downregulated signature associated with increased PD cases and is inversely correlated with UPDRS scores, suggesting a protective role in PD pathogenesis (Figure 6B).

The most correlated protein with UPDRS severity is angiopoietin-like 4 (ANGPTL4) (Pearson coefficient = 0.57), a lipid homeostasis modulator where elevated levels were recently associated with neurodegeneration (Supplementary Figure 3; Zhao et al., 2024). One possible mechanism is that hypoxic regions in the brain due to PD result in the upregulation of ANGPTL4 to compensate for this and modulate either inflammatory components or stress (González-Muniesa et al., 2011). Conversely, the most negatively correlated protein (Pearson coefficient = −0.56) is HPGDS, an enzyme that is involved in proteinoid synthesis and anti-inflammatory response (Ouyang et al., 2022). A possible relevance is that lower Hematopoietic prostaglandin D synthase (HPGDS) means less PGD2, leading to more unchecked neuroinflammation and worsening UPDRS scores (Corwin et al., 2018). Overall, feature clustering on the PPMI dataset shows strong protein signatures associated with PD cases and UPDRS scores. The high overlap between the cluster signature and the signature in the feature–feature correlation is possibly a core protein signature that can inform future studies to better understand PD mechanisms.

## 3 Discussion

Our PD prediction algorithms from plasma proteomics confirmed several established biomarkers as well as potentially novel biomarkers that warrant further investigation. The top features in both the ridge regression and neural network models are DDC and CALB2, which have previously been implicated in PD (Rutledge et al., 2024; Poulin et al., 2014). DDC is involved in dopamine synthesis (Pedersen et al., 2023) and is directly related to massive cell death of dopamine neurons in the striatum (Burkhard et al., 2001). CALB2 is heavily featured in a single-cell analysis of PD as a key marker for A10 dopamine neurons as compared to A9 dopamine neurons (Poulin et al., 2014). Ridge regression coefficients show CALB2 as negatively associated with PD, which is consistent with its expression in A10 mDA cells that are mostly spared from cell death during PD progression (Grealish et al., 2010).

In our study, several other less direct markers, such as hormonal components, were significantly correlated with PD incidence. These hormonal features, especially prolactin, were significantly correlated in multiple models and cluster analyses. Our findings illustrate a strong hormonal component, with 6 of the top 20 proteins identified by the neural network being hormonally related through heavy neurodevelopmental involvement and links to apoptotic functions. Further evidence from a protein interaction analysis identified these six proteins as closely functionally related (*p* < 6.15e-11; Figure 4B).

Another significant marker, human growth hormone (GH1), has several known functions ranging from growth of various tissues to neurogenesis (Martínez-Moreno and Arámburo, 2020). Notably, GH1 has been used to treat amyotrophic lateral sclerosis *in vitro* as well as in mouse models, demonstrating strong neurological protection (Chung et al., 2015). This is consistent with our findings, where GH1 is enriched in the control group relative to PD cases, making it a potentially protective marker. Prolactin produced by lactotrophs in the pituitary gland is another hormone that could be critical to understanding PD (Lamberts and Macleod, 1990). Prolactin (PRL) is known for its multifaceted impacts, including neuroinflammation, which is part of the pathogenesis of PD (Çınar et al., 2022), and modulation of PRL may offer a new therapeutic path for PD. Studies have also shown that prolactin release is regulated by dopamine (Binart et al., 2019), and therefore, loss of PRL may indicate loss of dopamine.

One common inflammatory model used to study neuroinflammation in mouse models is through intranasal lipopolysaccharide injections. This model led to selective dopamine substantia nigra (SN) loss and increased alpha synuclein in the SN (He et al., 2013). Furthermore, it was shown that in this lipopolysaccharide model, PRL is part of a full-autocrine loop that enhances inflammatory response that may contribute to further worsening PD through enhanced inflammation (López-Rincón et al., 2013).

PRL and GH1 play a strong role in the brain with widespread expression. This role especially extends past simply neurodevelopmental. PRL and GH1 are highly expressed in the cerebellum (Supplementary Figure 5A). Findings of cerebellar circuitry alteration in PD highlight the importance the cerebellum plays in PD progression and disease pathology (Li et al., 2023). The cerebellum is perhaps more involved in PD pathology than previously thought and may serve as an understudied area that contains biomarkers indicative of PD.

We performed a KEGG pathway enrichment analysis with the top 20 neural network features (see Methods). The core enriched pathways highlight the role of JAK–STAT and AKT pathways in PD pathogenesis. These are broad signaling pathways, but JAK2/STAT5 are commonly associated with developmental embryogenesis (Morris et al., 2018). These pathways are likely associated with PRL and GH1 activities, as both receptor bindings can lead to JAK2 activation (Morris et al., 2018; Supplementary Figure 5B).

While our study focused on PD, it represents a new framework to investigate other diseases through multiomic integration of proteomics and variants in the UK Biobank and other public data resources (Biobank, U, 2014; Denny et al., 2019; Nagai et al., 2017).

We have laid the groundwork to continue building on this multiomic model to provide an analysis framework to integrate proteomic Olink data and variant data in the UK Biobank. This approach can identify new associations with other neurodegenerative diseases. For example, through phenotype–gene enrichment analysis, we found several phenotypic PD features associated with height, which has some suggestive correlation with PD (Supplementary Figure 6; Saari et al., 2022; Ragonese et al., 2007). With an increase in Olink data in the UK Biobank, such new insights into the mechanisms of these diseases can now be investigated.

Plasma proteomics emerges as a powerful, minimally invasive alternative to cerebrospinal fluid-based biomarkers, achieving comparable classification performance while offering broader accessibility for large-scale screening. By leveraging machine learning models, we identified biomarkers, including hormonal markers such as prolactin and GH1, that suggest an underexplored hormonal axis in PD pathogenesis. Additionally, pathway analysis implicates the JAK–STAT and PI3K-AKT pathways in linking neuroinflammation, hormonal signaling, and neuronal survival. These findings not only demonstrate the feasibility of integrating plasma proteomics into cost-effective, population-level screening programs for early diagnosis and intervention but also provide a framework that can be extended to investigate biomarkers for other neurodegenerative diseases.

## 4 Materials and methods

### 4.1 Study population

The study population consisted of two groups from the UK Biobank cohort and Parkinson’s Progressive Marker Initiative cohort (Biobank, U, 2014; Marek et al., 2011). The UK Biobank is a large-scale biomedical database containing de-identified information of over 500,000 patients, including genotyping as well as serum analysis. Diagnosis of Parkinson’s disease was made by the International Classification of Diseases coded as ICD-9 and ICD-10 codes. Single-nucleotide polymorphisms were taken from the Axiom Array. The UK Biobank, after processing for missingness and filtering for Olink data, consisted of 43,408 patients: 23,329 were female and 20,079 were male, with a median age of 59 years. The PPMI dataset is an international consortium across 12 countries that is primarily sponsored by the Michael J. Fox Foundation (Marek et al., 2011). The PPMI uses two arrays for SNPs, including Immunochip and NeuroX, specially designed for PD. After processing, there were approximately 103 individuals, consisting of 29 females and 74 males, with a median age of 60 years. Diagnosis of PD was made with the addition of Dopamine Transporter Imaging (DAT) imaging to enhance accuracy (Marek et al., 2011).

We subset these datasets for participants that had Olink serum data collected. This substantially limited the number of participants. Both datasets used Olink Explore 1,536, so feature alignment was possible. A total of 1,463 proteins were measured from serum via the proximity extension assay (PEA), a high-multiplex immunoassay, after filtering. Olink Explore 1,536 contains 4 separate panels: oncology, cardiometabolic, inflammation, and neurology (Zannad et al., 2022). The assay works by double antibody binding to proteins leading to DNA hybridization, which can be PCR amplified, sequenced, and quantified. Proprietary normalization of these values provides protein quantification, allowing comparison across individuals and studies.

### 4.2 Variant preprocessing and alignment

Genomic variants were preprocessed using PLINK 2.0 software, a genome association toolkit (Chang et al., 2015). We aligned the variants in both arrays by position, major allele, and minor allele to Genome Reference Consortium Human Build 37. The UK Biobank used the Axiom array, while the PPMI dataset used the Immunochip and NeuroX arrays. We filtered for the top 324 overlapping variants and used an additional 311 variants identified in Kim et al. (2024). These were taken from the Polygenic Score (PGS) catalog, as these were previously found to have signal in a non-publicly accessible 23andMe dataset with *p* < 1.35E-03 (Lambert et al., 2021). This proprietary dataset allowed for more power, leading to the identification of associated variants. In total, 635 filtered variants with overlap across both the UK Biobank and PPMI validation set were used in our prediction models.

Several QC steps were performed at the variant level. We applied a series of standard filters to ensure data quality and reliability. We excluded variants that did not conform to the Hardy–Weinberg equilibrium, applying a *p*-value threshold of < 1e-6 to maintain population consistency. Variants with a call rate below 95% were filtered out due to missing data concerns, while ambiguous SNPs (A/T or C/G alleles) were removed to avoid strand ambiguity. A minor allele frequency (MAF) threshold of >1% was set to focus on variants with sufficient population representation. Additionally, we filtered variants based on imputation quality, using an Imputation Info score > 0.8 to ensure high-confidence imputation calls. Relatedness was assessed using a kinship coefficient, excluding individuals with relationships stronger than first-degree (>0.125). Finally, we removed samples exhibiting sex discordancy between self-reported and genotypic sex to ensure consistency in a sex-based genetic analysis.

### 4.3 Study design

The UK Biobank was used for training and testing, and PPMI data were used as an independent external test to measure classifier performance. Patients were filtered for European ancestry and missing features. In addition, features were filtered for missing values. The resulting multiomic dataset combined demographic data, genomic data, and proteomic data. We used several baseline models, such as a ridge regression model, which performs L2 penalization to minimize coefficients (Wu and Xu, 2020). Additionally, an SVM model with a linear kernel was used as a second baseline model. For training and testing, a split of 60 and 40% was used, respectively. The ratio of PD cases to control was maintained in training and test splits. We chose a 60 and 40% split for training and testing to balance a robust training process with a large and independent test set. A larger training set allows the model to learn more generalizable patterns from the data, which is especially important for complex tasks such as disease classification. Given the small number of PD cases in our dataset, using 60% of the data for training maximized the number of cases for the model to learn while still having a large enough test set to give reliable performance metrics. By using the same 60 or 40% split across different models (e.g., SNP-only model, ridge regression model, and SVM model), we were able to compare their performance on the same datasets and ensure consistency and fairness in model evaluation. The genetic single-nucleotide polymorphism-only model used the same split of 60 and 40% for the training and test dataset. Ridge regression and SVM were tuned by grid search to identify the best model. Both used class weighting to address imbalance common with medical data.

The neural network architecture is a hidden two-layer neural network. In this fully connected neural network, the size of the first hidden layer is 120, and the size of the second hidden layer is 40. Activation was done by leaky ReLU. A sigmoid function was then applied for the final output layer binary prediction value. The model uses binary cross entropy (BCE) as the loss function that assigns a larger error for mislabeled cases as compared to mean square loss, which is more appropriate for training data with a relatively low fraction of positive cases.


$$\mathrm{BCE}=-\frac{1}{N}\sum_{i=1}^{N}[y_{i}\log(p_{i})+(1-y_{i})\log(1-p_{i})]$$

where N is the number of samples, yi is the label (i.e., PD or control), and pi is the predicted probability of yi. Several steps were taken to deal with imbalanced cases, including class weights and model architecture adjustments. Dropout was used to address overfitting, which was emboldened by imbalanced cases. Furthermore, a large batch size was used to give more positive PD cases for each stochastic gradient descent step using the Adam optimizer. The neural network architecture and training parameters were selected through a grid search, which identified two hidden layers with 120 and 40 units, respectively, a dropout rate of 0.2, a learning rate of 0.001, a weight decay of 1e-4, and a training duration of 40 epochs as the optimal configuration.

### 4.4 Feature selection and predicting UPDRS scores

A linear model was built to associate the common predictive features from the neural network and ridge regression with the UPDRS severity score. Using an increasing number of common features (from 20 to 200), we found 85 features with optimal R^2^ value and minimized overfitting (Supplementary Figure 2).

The samples were clustered using these 85 features by the K-means approach, with *K* = 3, as determined by a silhouette analysis (Supplementary Figure 7).

### 4.5 Feature correlation analysis

A feature–feature correlation analysis was performed on these top 85 features using Pearson’s correlation. The UPDRS score was included in these correlation calculations and hierarchical clustering of the correlation values (Supplementary Figure 3).

### 4.6 Model interpretation

Feature weights on the ridge regression model were set through the coefficients placed on the optimized model. The neural network used a game-theoretic approach to feature importance called SHAP scores (Lundberg and Lee, 2017). These scores create a power set of features to determine the marginal contribution of each feature. The mean absolute SHAP scores were taken across all combinations of models on the testing set to determine the feature importance. The distribution was visualized through a bee swarm plot. Additionally, a phenotype–gene enrichment analysis was performed to detect statistically significant phenotypes from our top 20 features. The top 20 features were run for feature enrichment analysis. Ultimately, the results for top KEGG phenotypes were similar across 20–100 feature combinations. To focus on the most impactful features, we used the top 20 and highlighted these using SHAP scores and KEGG analyses.

### 4.7 KEGG analysis

For the KEGG pathway analysis, we used an overrepresentation analysis with hypergeometric tests to find the pathways associated with the features selected from our models. KEGG pathway analyses were conducted using two sets of features: the top 20 features identified by our neural network model and a broader subset of 85 features jointly selected from both the neural network and ridge regression models. These 85 features represented the most predictive markers shared across models. This approach allowed us to identify enriched pathways that are both statistically significant and mechanistically relevant to Parkinson’s disease pathology. This way we capture a wider range of pathways while maintaining enough statistical power to interpret the results.

### 4.8 Limitations

Several limitations are present, including GH1 levels that are known to decrease with age (known as somatopause) and PRL levels that are typically higher in women and inhibited by dopamine (Ben-Jonathan and Hnasko, 2001; Camanni et al., 1977; Reyes et al., 1977). These factors represent significant confounders that must be considered in the analysis, although they were mitigated as best as possible. In addition, while we demonstrated that prior L-dopa treatment had minimal impact on our model performance, we cannot exclude the possibility that with more detailed medical history, L-dopa and other treatments can emerge as confounders.

Additionally, age- and endocrine-related confounding were not fully controlled in the current analyses, and, therefore, the observed associations for GH1 and PRL should be interpreted with caution. While cohort-level sex distributions differed between the UK Biobank and the PPMI, sex and age were included as covariates, suggesting robustness to these differences. Future studies with tighter age and gender stratification are possible in the future with a larger caseload, as there was already a heavy class imbalance we were concerned with.

Olink also has a problem of hemolysis, which is the rupture of red blood cells during testing, which can cause intracellular proteins to leak out to plasma and bias results.

In addition, batch effects exist from the UK Biobank to the PPMI dataset. Blood was collected in different facilities, although with the same assay and normalization process. Furthermore, the UK Biobank is a population-level study, while the PPMI is a hand-selected longitudinal PD patient population, creating different underlying distributions.

## Data Availability

The UK Biobank and PPMI genomic and phenotypic data is restricted and requires prior approval for access and therefore cannot be provided in this publication. Requests to access these datasets should be directed to https://www.ukbiobank.ac.uk/; https://www.ppmi-info.org/.
