# Supplementary material for: Machine learning analysis of population-wide plasma proteins identifies hormonal biomarkers of Parkinson’s disease
Source: Front Aging Neurosci. 2026 Mar 10;18:1730550. doi: 10.3389/fnagi.2026.1730550 (PMC13008742; doi:10.3389/fnagi.2026.1730550)
Supplement: Supplementary file 1 [file Supplementary_file_1.pdf]

Supplementary Material

Supplement Table 1: Performances of PD prediction models using genetic variants and demographic data. Like table 1, the performance of the three models using SNPs variants was evaluated by AUC, sensitivity, specificity measures for PD classification performance on the UK biobank held-out data as well as AUC performance on the PPMI external validation set.

	Ridge Regression	SVM	Neural Network
AUCROC Test Set (UK Biobank)	0.70	0.70	0.62
Sensitivity	0.72	0.59	0.37
Specificity	0.55	0.68	0.78
AUCROC Validation Set (PPMI)	0.55	0.45	0.51

Classification Performance without L-Dopa Treated Patients

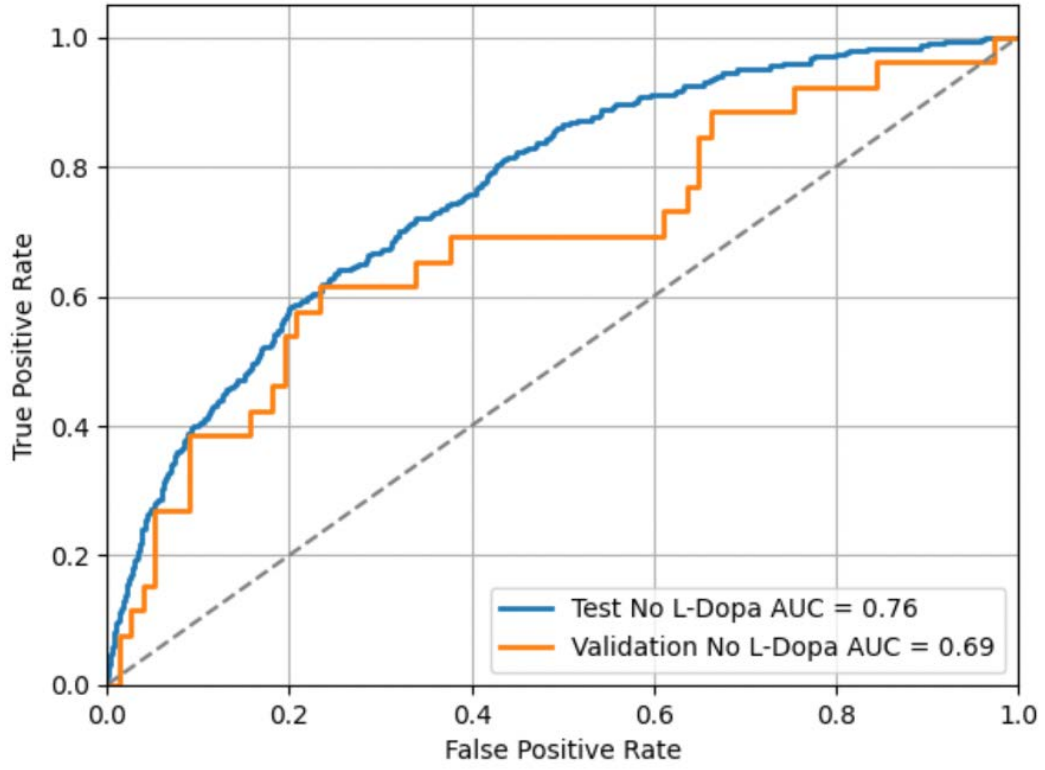

Supplementary Figure 1: ROC curves comparing model performance of the ridge regression on test and validation sets after L-Dopa ablation. The test set achieved an AUC of 0.76, while the validation set reached an AUC of 0.69, indicating moderately reduced generalization. These results suggest that excluding L-Dopa–associated features impact predictive accuracy but retains meaningful signal.

Top Feature Coefficients in PD Severity Model

Feature	Coefficient	Feature	Coefficient
SCG3	14.5826	CA13	-0.3346
TNFRSF12A	14.0563	CD276	-0.3788
DSG3	13.5694	FOXO3	-0.4835
X4.1312394_C_T_T	12.5281	SMARCA2	-0.6183
ADAMTS8	10.734	MAX	-0.6533
NTF4	8.1926	SIRPA	-0.6547
CES2	7.7873	PAEP	-0.7416
THBS4	7.6361	PI3	-0.8877
ADA2	6.8871	X2.135443940_A_G_G	-0.9686
X4.114369065_C_T_T	6.3448	MOG	-1.0727
GZMB	6.2542	RRM2	-1.8321
DKK4	6.2251	CPPED1	-1.8941
FABP5	5.6416	CASP10	-2.108
IL15RA	5.2286	EDA2R	-2.2455
PADI2	5.115	PLA2G2A	-2.6745
SCLY	4.854	IMPA1	-2.701
CA14	4.7165	GDF15	-2.7445
ATG4A	4.2999	CRACR2A	-2.8543
USO1	3.9872	X7.129663496_C_T_T	-2.8699
ANGPTL4	3.7123	CCL27	-2.955
CCL17	3.4713	NCAM1	-3.5072
IL11	2.8768	NCF2	-4.0899
ARG1	2.6362	ITGA11	-4.1488
PRL	2.4548	CCN5	-4.1616
CCL25	2.4422	CEACAM3	-4.3656
DPEP1	2.4298	ACE2	-4.5744
PRDX1	2.2077	FABP9	-4.775
SPP1	2.025	KLK8	-5.4758
DDC	1.82	CLEC10A	-5.596
BAG3	1.8191	X16.11231857_G_A_A	-5.8894
SFTPD	1.7606	PTPRN2	-6.2858
THBS2	1.7517	CTSB	-6.6593
NCS1	1.6994	CDH6	-6.9729
X17.40741013_T_C_C	1.5345	IRAK4	-7.1918
CTSV	1.0043	CA3	-9.0615
CCL13	0.8506	ICAM4	-9.4953
X7.23430418_C_T_T	0.6096	ITGB7	-9.6421
NEFL	0.3588	ANGPTL7	-13.0296
HNMT	0.1684	SCG2	-13.685
CALB2	0.1453	X10.104015279_A_G_G	-14.746
CXCL13	0.0986	HPGDS	-16.9367
CDC27	0.0842	DPT	-18.697

Supplementary Table 2: Feature coefficients ranked by magnitude, representing their contribution to the predictive model. Positive coefficients indicate features positively associated with the target outcome, while negative coefficients represent inverse associations. This ranking highlights biomarkers with potential mechanistic or diagnostic relevance.

Effect of Number of Features on Severity Prediction

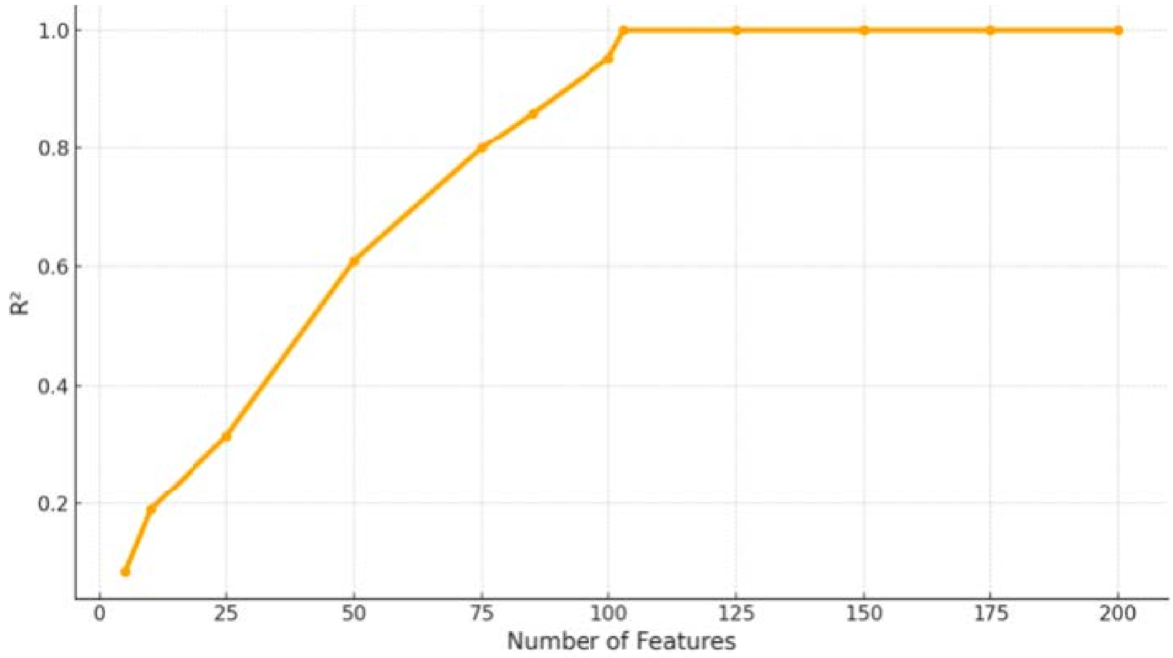

Supplementary Figure 2: The plot shows how the number of input features, ranging from 5 to 250, affects the R^2 score in a regression model predicting the UPDRS score. As the number of features increases, the model performance improves, indicating a positive correlation between feature count and predictive accuracy.

Features and UPDRS Correlations

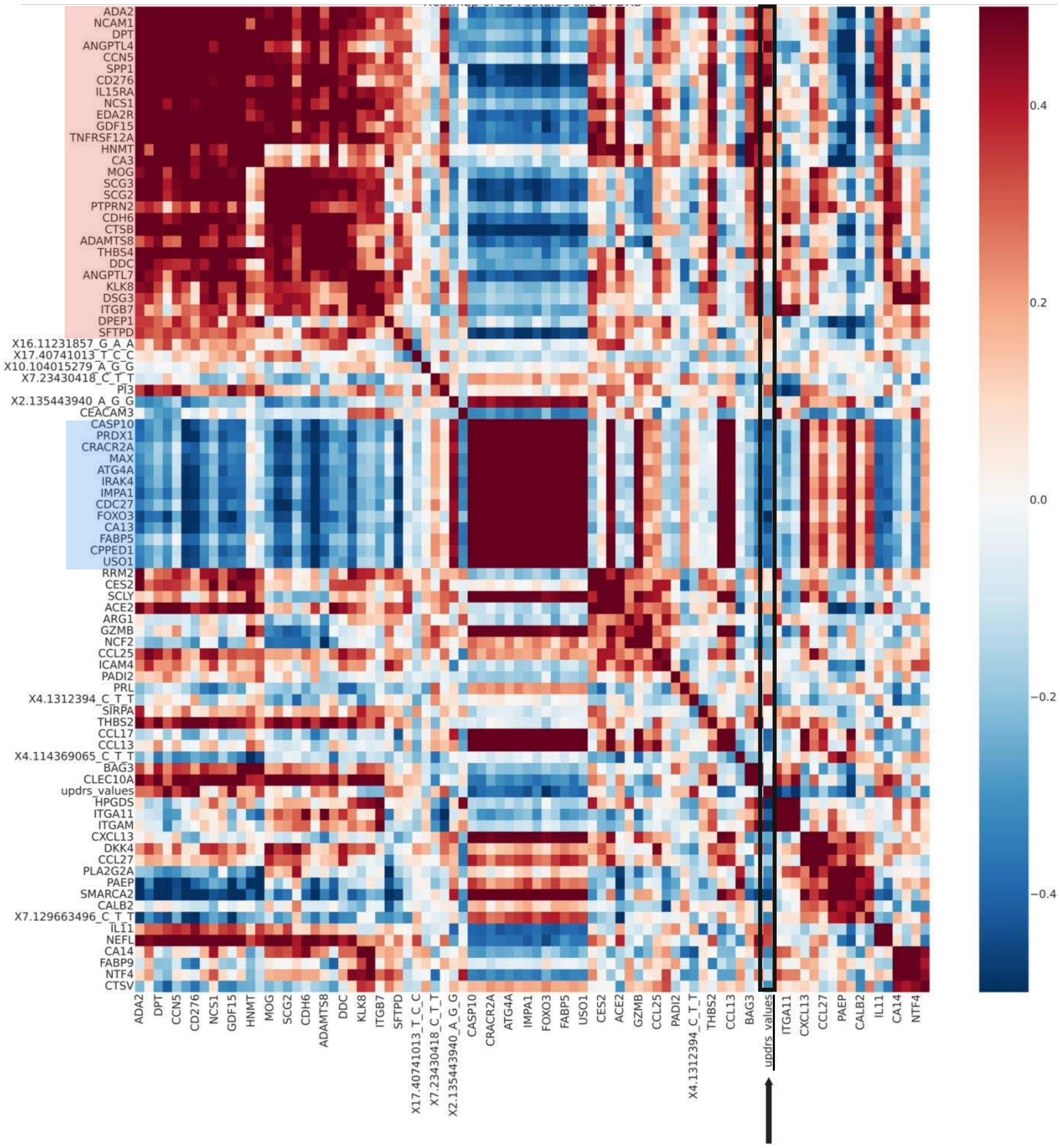

Supplement Figure 3: Feature correlation. The Pearson correlation heatmap between 85 selected features and the UPDRS score, with color intensity indicating the strength and direction of the correlations. Strong correlations are marked by darker shades, while lighter shades represent weaker correlations, highlighting potential patterns and associations relevant to Parkinson's severity. Two main feature groups are strongly correlated (in red) and anticorrelated (in blue) with UPDRS scores (marked by black arrow). Note that due to space constrains every second feature is labeled on the vertical axis.

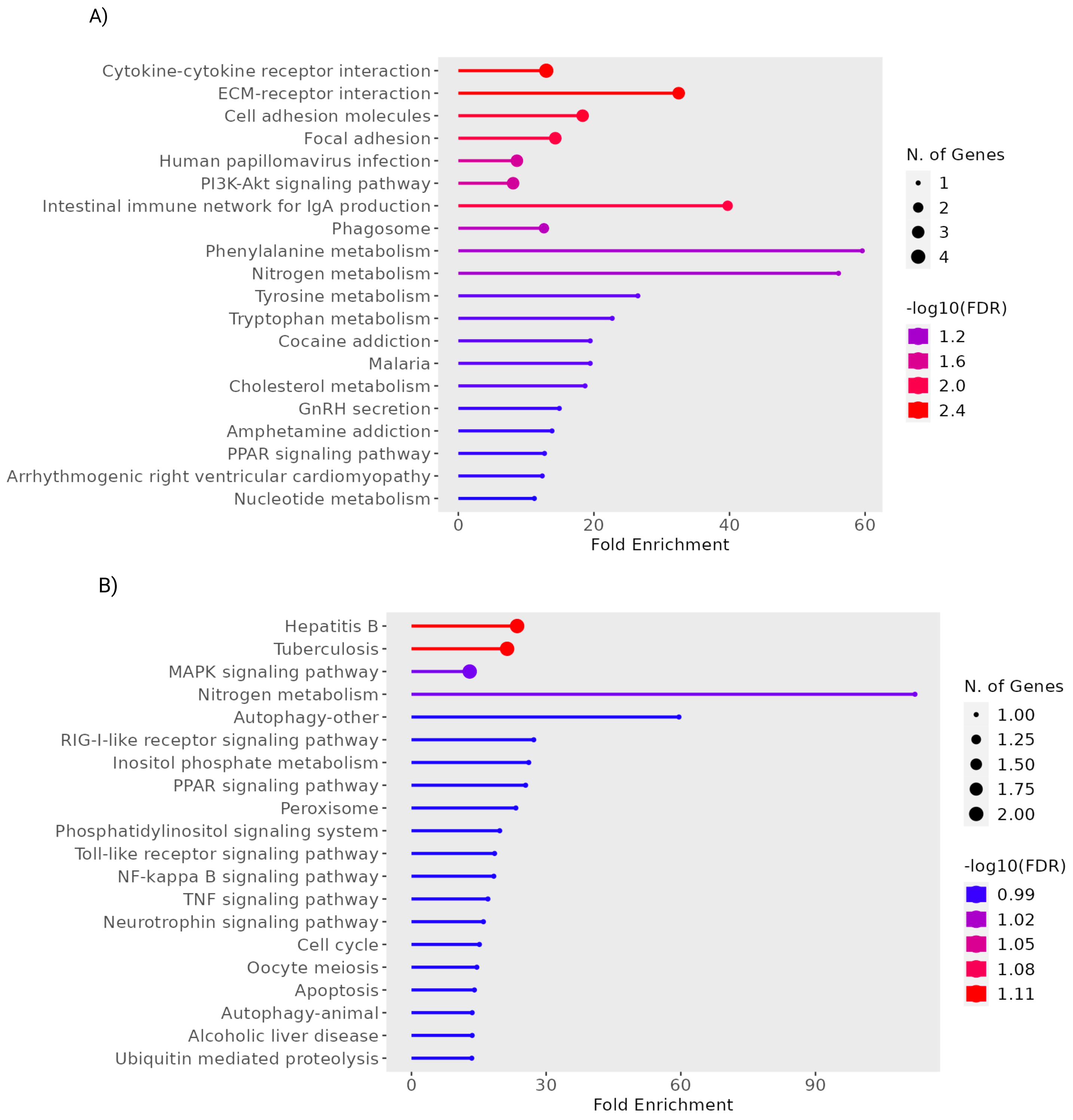

Supplementary Figure 4: Pathway enrichment of UPDRS correlated features. A) KEGG analysis of top UPDRS correlated features (red in group in Supplementary Figure 1) highlight enrichment in the cytokine-cytokine receptor interaction and PI3K-AKT signaling pathways. B) Similarly, enrichment analysis of top UPDRS anticorrelated features highlights the MAPK signaling pathway and apoptotic pathways.

A

B

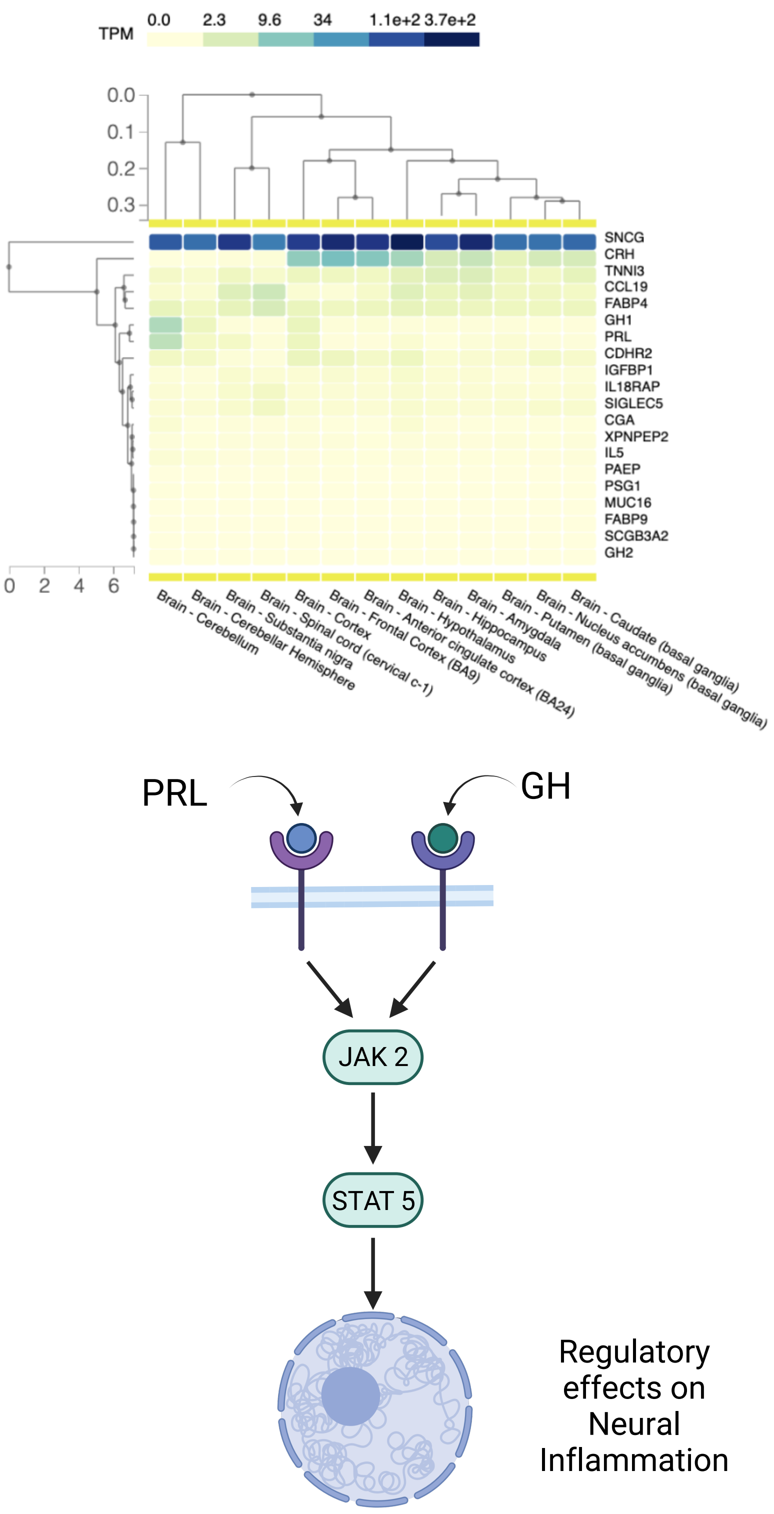

Supplementary figure 5: (A) Gene expression levels across different brain tissues from the GTEx study [50], highlighting the differential expression patterns relevant to the pathway including, GH1 and PRL expression in the Cerebellum. (B) The PRL pathway depicting the JAK2-STAT5 signaling cascade, illustrating key molecular interactions and activation steps.

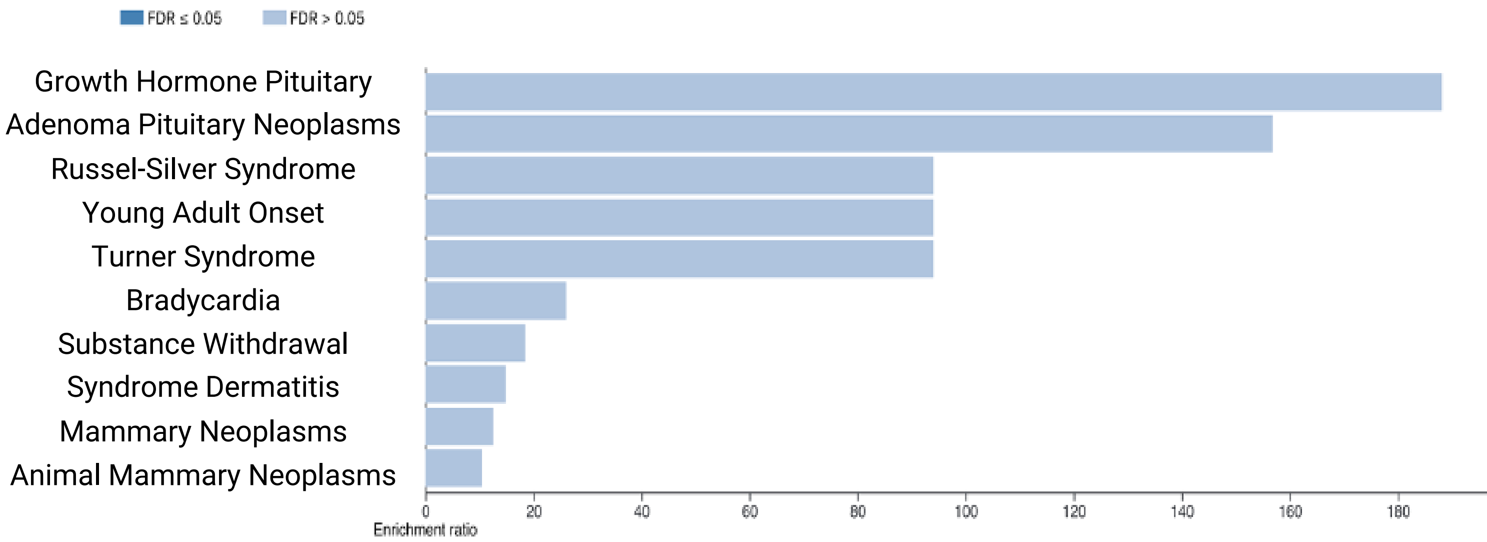

Supplementary Figure 6: Phenotype-gene enrichment analysis of the top 20 features identified by the neural network model, conducted using the WEB-based GEne SeT AnaLysis Toolkit. The enrichment analysis highlights significant associations between these features and relevant gene sets, providing insights into potential biological pathways linked to the observed phenotypes.

Silhouette Score by Number of Clusters

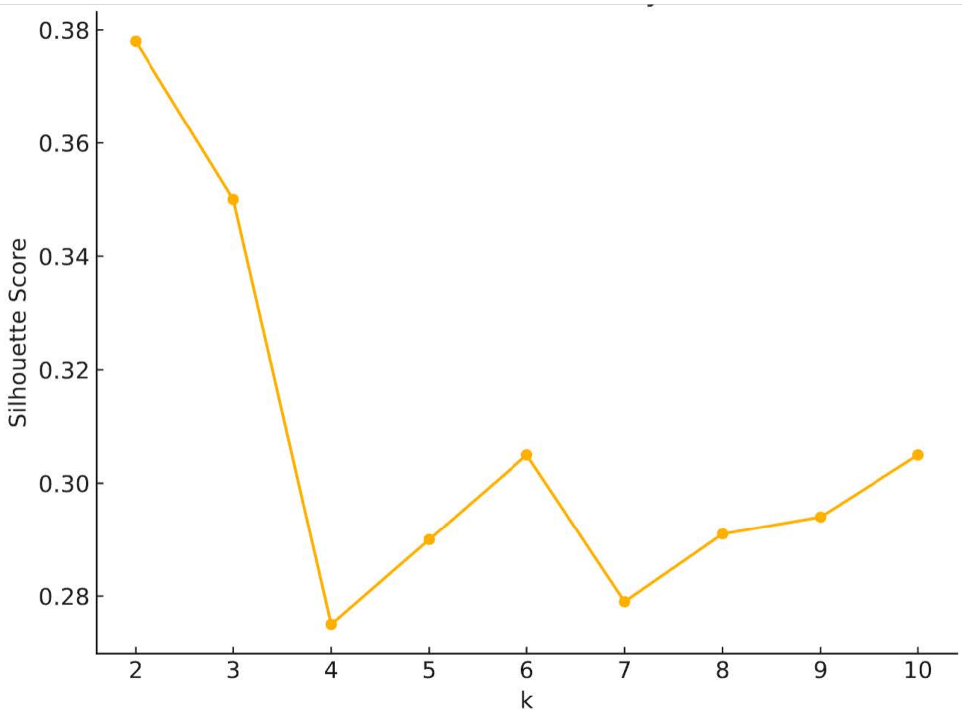

Supplementary Figure 7: Silhouette Score plotted against different values of k for clustering analysis. The graph indicates a sharp decrease in score after k = 3. Based on this trend and domain-specific considerations, k = 3 was selected as the optimal number of clusters, balancing interpretability with separation quality..
